# Pain modulatory network is influenced by sex and age in a healthy state and during osteoarthritis progression in rats

**DOI:** 10.1111/acel.13292

**Published:** 2021-01-05

**Authors:** Joyce T. Da Silva, Christina Tricou, Youping Zhang, Amir Tofighbakhsh, David A. Seminowicz, Jin Y. Ro

**Affiliations:** ^1^ Department of Neural and Pain Sciences School of Dentistry University of Maryland Baltimore Baltimore Maryland USA; ^2^ Center to Advance Chronic Pain Research University of Maryland Baltimore Baltimore Maryland USA; ^3^ Department of Psychiatry School of Medicine Johns Hopkins University Baltimore Maryland USA

**Keywords:** aging, brain, osteoarthritis, pain, pain inhibition, sex

## Abstract

Old age and female sex are risk factors for the development of osteoarthritis (OA) and chronic pain. We investigated the effects of sex and age on pain modulatory networks in a healthy state and during OA progression. We used functional MRI to determine the effects of sex and age on periaqueductal gray functional connectivity (PAG FC) in a healthy state (pre‐OA) and during the early and late phases of monosodium iodoacetate‐induced OA in rats. We then examined how sex and age affect longitudinal changes in PAG FC in OA. In a healthy state, females exhibited more widespread PAG FC than males, and this effect was exaggerated with aging. Young males had moderate PAG FC changes during the early phase but recruited additional brain regions, including the rostral anterior cingulate cortex (ACC), during the late phase. Young females exhibited widespread PAG FC in the early phase, which includes connections to insula, caudal ACC, and nucleus accumbens (NAc). Older groups had strong PAG FC with fewer regions in the early phase, but they recruited additional brain regions, including NAc, in the late phase. Overall, our findings show that PAG FC is modulated by sex and age in a healthy state. A widespread PAG network in the early phase of OA pain may contribute to the transition from acute to chronic OA pain and the increased risk of developing chronic pain for females. Enhanced PAG FC with the reward system may represent a potential mechanism underlying chronic OA pain in elderly patients.

## Graphical abstract

In a healthy state, females exhibit widespread periaqueductal gray functional connectivity (PAG FC), and this effect is exaggerated with aging. Young females have widespread PAG network in the early phase of osteoarthritis (OA) pain. PAG FC with rostral anterior cingulate cortex in young males in the late OA phase may facilitate descending pain inhibition. Enhanced PAG FC with the reward system is seen in old groups in the late OA phase.

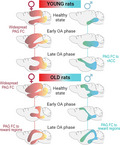

## 1 INTRODUCTION

Osteoarthritis (OA) is a leading cause of chronic pain and disability and a major public health problem due to its increasing prevalence with an aging population (Dieppe & Lohmander, 2005). In addition, women exhibit higher prevalence of OA and associated pain disability than men (Parmelee et al., 2012). The high OA prevalence in the elderly does not reflect a simple consequence of the biological changes associated with age but rather the complex interplay between age, sex, and pain processing at multiple levels of the neuroaxis (Cottam et al., 2016; Keszthelyi et al., 2018). Pain in OA patients is associated with abnormal activation of widespread brain regions including somatosensory cortex, insula, anterior cingulate cortex (ACC), thalamus, amygdala, nucleus accumbens (NAc), hippocampus, and the periaqueductal gray (PAG), all of which are involved in pain processing, reward, and emotion (Howard et al., 2012; Kulkarni et al., 2007). PAG and ACC, along with the rostral ventromedial medulla, form a functional core of the descending pain modulatory network (Chen et al., 2015; Fields, 2004; Kong et al., 2010). Functional connectivity (FC) alterations between PAG and the rostral ACC (rACC) are consistently found in patients with OA pain (Chen et al., 2015). A preclinical study using intra‐articular injection of monosodium iodoacetate (MIA) as an OA pain model also supported the role of PAG dysregulation in OA (Abaei et al., 2016). It is currently hypothesized that alterations in descending pain modulatory networks may contribute to the pathophysiological basis for chronic OA pain, and potential therapies could target these networks (Cottam et al., 2016; Keszthelyi et al., 2018). However, the influence of sex and age on descending pain modulatory networks remains poorly investigated, not only in OA, but also in a healthy state.

We have previously shown that diffuse noxious inhibitory control (DNIC), also known as the pain‐inhibits‐pain phenomenon, is more efficient in healthy young male (YM) than healthy young female (YF) rats (Da Silva et al., 2020). In addition, DNIC response is lost in healthy rats of both sexes as they age, and aging is associated with alterations in brain connectivity, including between PAG and ACC (Da Silva et al., 2020). It is likely that differences in descending pain modulatory networks in a healthy state may contribute to the increased risk of developing chronic pain for females and the higher susceptibility to pain modulatory deficits in the elderly population (Fillingim, 2000; González‐Roldán et al., 2020).

Another important question that remains unaddressed in the OA field is how brain changes occur during OA pain progression. The MIA model allows us to investigate two distinct phases of OA pain behavior (Bove et al., 2003). The early phase (0–10 days) is associated with hypersensitivity mostly driven by the initial inflammatory phase, whereas the late phase (14–28 days) is associated with established pain behaviors and the progression of subchondral bone lesions that mimic those observed in OA patients (Bove et al., 2003; Mapp et al., 2013). Using the MIA‐induced OA model in Fisher rats, we reported that hyperalgesia lasts longer and is more pronounced in older rats, with aged female rats showing the most impaired responses (Ro et al., 2020). This data are consistent with human studies and suggest that age significantly impacts OA‐like pain, making the elderly, particularly females, more vulnerable to chronic OA pain (Felson et al., 1987; Shane Anderson & Loeser, 2010).

In this study, we sought to determine the effects of sex and age on PAG FC in a healthy state and in the early and late phases of MIA‐induced OA. We then examined the longitudinal changes in PAG FC from early to late phases of the MIA‐induced OA model in rats stratified by sex and age. We hypothesized that PAG FC is influenced by sex and age under healthy and OA conditions and that alterations in PAG FC are further impacted as OA progresses from acute to chronic states.

## 2 RESULTS

### 2.1 Sex and age differences in FC of PAG in a healthy state

**FIGURE 1:**
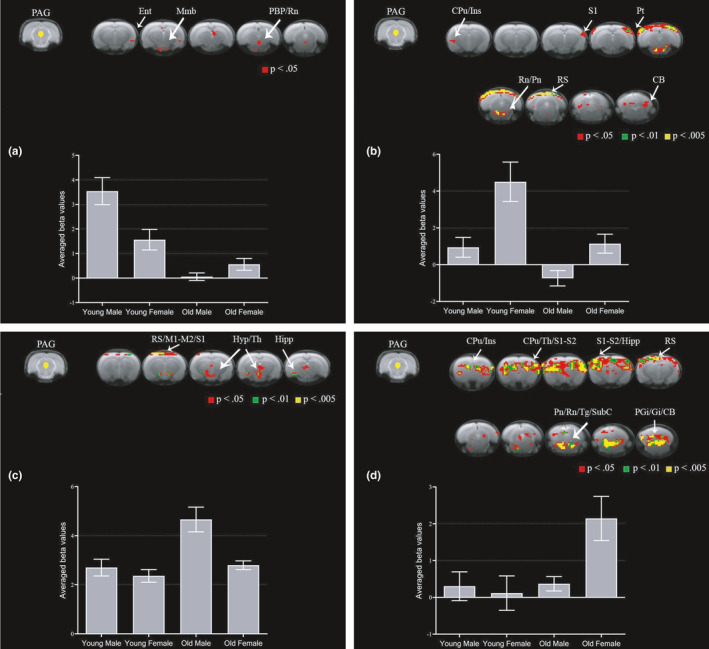
Sex and age differences in PAG connectivity to the whole brain at baseline. Group comparisons showing increased connectivity between PAG and brain regions in young males (a), young females (b), old males (c), and old females (d). Brain template shows cluster‐forming thresholds at *p* < 0.05, 0.01, and 0.005. Plots show extracted beta values from all significant clusters with a forming threshold of *p* < 0.05 for each group (average ±S.E.M.). CB, cerebellum; CPu, caudate putamen; Ent, entorhinal cortex; Gi, gigantocellular reticular nucleus; Hipp, hippocampus; Hyp, hypothalamus; Ins, insula; M1, primary motor cortex; M2, secondary motor cortex; Mmb, mammillary body; PAG, periaqueductal gray; PBP, parabrachial pigmented nucleus of the ventral tegmental area; PGi, paragigantocellular nucleus; Pn, pontine nuclei; Pt, parietal cortex; Rn, raphe nuclei; RS, retrosplenial cortex; S1, primary somatosensory cortex; SubC, subcoeruleus nucleus; Tg, tegmental nucleus; Th, thalamus

We first assessed sex and age differences in PAG FC with the whole brain at baseline since sex and age have been shown to be key factors affecting brain connectivity in a healthy state (Da Silva et al., 2020; González‐Roldán et al., 2020; Kong et al., 2010). Young male rats had increased PAG FC with entorhinal cortex, mammillary body, parabrachial pigmented nucleus of the ventral tegmental area, and raphe nuclei relative to YF, old male (OM), and old female (OF) rats (Figure 1a). Young female rats had increased PAG FC with caudate putamen, insula, primary somatosensory cortex, parietal cortex, raphe nuclei, pontine nuclei, retrosplenial cortex, and cerebellum relative to YM, OM, and OF rats (Figure 1b). OM rats had increased PAG FC with retrosplenial cortex, primary and secondary motor cortices, primary somatosensory cortex, hypothalamus, thalamus and hippocampus relative to YM, YF, and OF rats (Figure 1c). OF rats had increased PAG FC with caudate putamen, insula, thalamus, primary and secondary somatosensory cortices, hippocampus, retrosplenial cortex, pontine nuclei, raphe nuclei, tegmental nucleus, subcoeruleus nucleus, paragigantocellular nucleus, gigantocellular reticular nucleus, and cerebellum relative to YM, YF, and OM rats (Figure 1d). These findings show that old rats have more widespread PAG FC with the whole brain at baseline compared to young rats of the same sex (see also Figure S2). However, female sex appears to contribute to the most widespread PAG connectivity pattern at baseline (see also Figure S1). YM display minimal group differences in PAG FC mapping, while YF have increased PAG FC, mainly with cortical regions including insula and primary somatosensory and parietal cortices. Thus, PAG connectivity pattern appears to be influenced by sex and age in a healthy state. We then decided to investigate sex‐ and age‐specific changes in PAG FC during the course of MIA‐induced OA.

### 2.2 Sex‐ and age‐dependent changes in FC of PAG in the early phase of MIA‐induced OA

**FIGURE 2:**
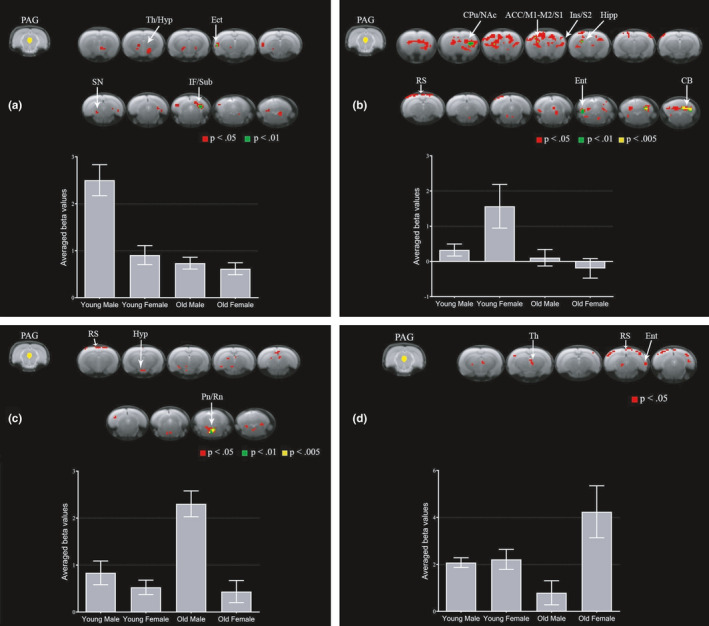
Sex and age differences in PAG connectivity to the whole brain in the early phase of MIA‐induced OA. Group comparisons showing increased connectivity between PAG and brain regions in young males (a), young females (b), old males (c), and old females (d). Brain template shows cluster‐forming thresholds at *p* < 0.05, 0.01, and 0.005. Plots show extracted beta values from all significant clusters with a forming threshold of *p* < 0.05 for each group (average ±S.E.M.). ACC, anterior cingulate cortex; CB, cerebellum; CPu, caudate putamen; Ect, ectorhinal cortex; Ent, entorhinal cortex; Hipp, hippocampus; Hyp, hypothalamus; IF, inferior colliculus; M1, primary motor cortex; M2, secondary motor cortex; NAc, nucleus accumbens; PAG, periaqueductal gray; Pn, pontine nuclei; Rn, raphe nuclei; RS, retrosplenial cortex; S1, primary somatosensory cortex; S2, secondary somatosensory cortex; SN, substantia nigra; Sub, subiculum; Th, thalamus

PAG FC is modulated by sex and age at baseline, and OA pain alters PAG FC and activity in humans and animals (Abaei et al., 2016; Chen et al., 2015). Furthermore, the progression of OA pain behavior over time is also modulated by sex and age in rats (Ro et al., 2020). Thus, we first decided to investigate the impact of sex and age on OA‐induced alterations in PAG FC during the early phase of MIA‐induced OA. YM rats had increased PAG FC with thalamus, hypothalamus, ectorhinal cortex, substantia nigra, inferior colliculus, and subiculum relative to YF, OM, and OF rats (Figure 2a). YF rats had increased PAG FC with caudate putamen, NAc, caudal ACC (cACC), primary and secondary motor cortices, primary and secondary somatosensory cortices, insula, hippocampus, retrosplenial cortex, entorhinal cortex, and cerebellum relative to YM, OM and OF rats (Figure 2b). OM rats had increased PAG FC with retrosplenial cortex, hypothalamus, pontine nuclei, and raphe nuclei relative to YM, YF, and OF rats (Figure 2c). OF rats had increased PAG FC with thalamus, retrosplenial cortex, and entorhinal cortex relative to YM, YF, and OM rats (Figure 2d). Group differences in PAG FC with the whole brain were statistically modest in the early phase, possibly due to the high pain behavior observed in all groups at this time point (Ro et al., 2020). However, YF had increased PAG FC with several brain regions compared to other groups, such as basal ganglia, NAc, cACC, and motor and somatosensory cortical areas. Overall, YF appear to recruit a more widespread PAG network in the early phase of OA compared to YM and older groups, which may be associated with the females’ increased risk of developing chronic pain conditions.

### 2.3 Sex‐ and age‐dependent changes in FC of PAG in the late phase of MIA‐induced OA

**FIGURE 3:**
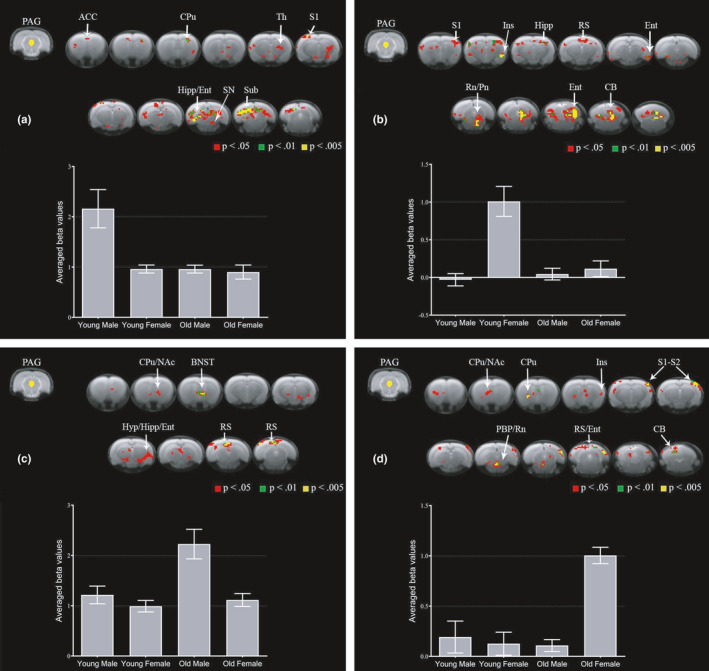
Sex and age differences in PAG connectivity to the whole brain in the late phase of MIA‐induced OA. Group comparisons showing increased connectivity between PAG and brain regions in young males (a), young females (b), old males (c), and old females (d). Brain template shows cluster‐forming thresholds at *p* < 0.05, 0.01 and 0.005. Plots show extracted beta values from all significant clusters with a forming threshold of *p* < 0.05 for each group (average ±S.E.M.). ACC, anterior cingulate cortex; BNST, bed nucleus of the stria terminalis; CB, cerebellum; CPu, caudate putamen; Ent, entorhinal cortex; Hipp, hippocampus; Hyp, hypothalamus; Ins, insula; NAc, nucleus accumbens; PAG, periaqueductal gray; PBP, parabrachial pigmented nucleus of the ventral tegmental area; Pn, pontine nuclei; Rn, raphe nuclei; RS, retrosplenial cortex; S1, primary somatosensory cortex; S2, secondary somatosensory cortex; SN, substantia nigra; Sub, subiculum; Th, thalamus

Next, we investigated the impact of sex and age on OA‐induced alterations in PAG FC in the late phase of MIA‐induced OA. YM rats had increased PAG FC with rACC, caudate putamen, thalamus, primary somatosensory cortex, hippocampus, entorhinal cortex, substantia nigra, and subiculum relative to YF, OM, and OF rats (Figure 3a). YF rats had increased PAG FC with primary somatosensory cortex, insula, hippocampus, retrosplenial cortex, entorhinal cortex, raphe nuclei, pontine nuclei, and cerebellum relative to YM, OM, and OF rats (Figure 3b). OM rats had increased PAG FC with caudate putamen, NAc, bed nucleus of the stria terminalis, hypothalamus, hippocampus, entorhinal cortex, and retrosplenial cortex relative to YM, YF, and OF rats (Figure 3c). OF rats had increased PAG FC with caudate putamen, NAc, insula, primary and secondary somatosensory cortices, parabrachial pigmented nucleus of the ventral tegmental area, raphe nuclei, retrosplenial cortex, entorhinal cortex, and cerebellum relative to YM, YF, and OM rats (Figure 3d). Notably, YM showed enhanced PAG FC with rACC, which may demonstrate a strong endogenous inhibitory capacity and a potential mechanism underlying the full recovery from OA‐induced hyperalgesia at this time point (Da Silva et al., 2020; Ro et al., 2020). Females, regardless of age, showed increased PAG FC with raphe nuclei (see also Figure S1). Males, regardless of age, showed enhanced PAG FC with the hippocampus (see also Figure S1). However, the specific regions of the hippocampus differed between YM and OM. Increased connectivity to NAc was observed only in older groups (see also Figure S2), which have been shown to exhibit persistent OA‐induced hyperalgesia at this time point (Ro et al., 2020).

### 2.4 Enhanced FC of PAG in the early phase of MIA‐induced OA in groups stratified by sex and age

**FIGURE 4:**
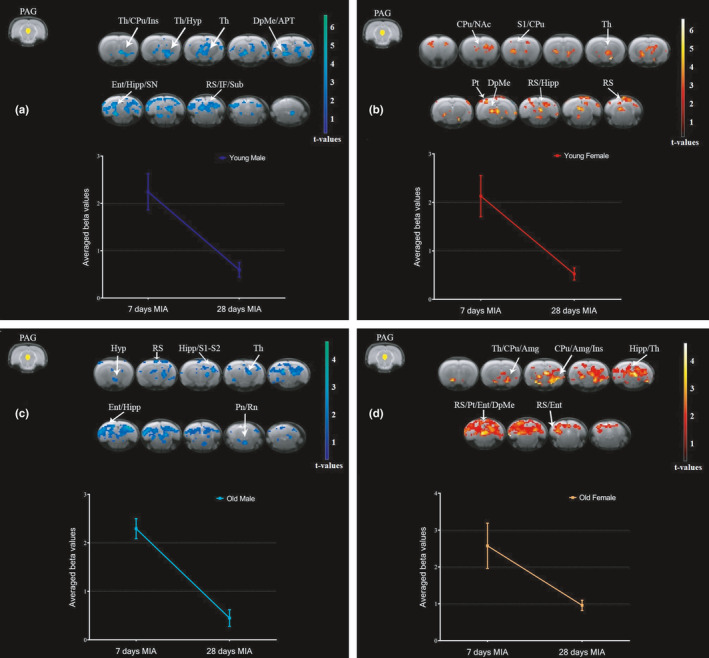
Increased PAG connectivity to the whole brain in the early phase of MIA‐induced OA in groups stratified by sex and age. Increased connectivity between PAG and brain regions at 7 days compared to 28 days after MIA injection in young males (a), young females (b), old males (c), and old females (d). We used a cluster‐forming threshold of *p* < 0.05, and t‐values are represented using the color‐coded scales. Plots show extracted beta values from all significant clusters for each group (average ±S.E.M.). Amg, amygdala; APT, anterior pretectal nucleus; CPu, caudate putamen; DpMe, deep mesencephalic nucleus; Ent, entorhinal cortex; Hipp, hippocampus; Hyp, hypothalamus; IF, inferior colliculus; Ins, insula; NAc, nucleus accumbens; PAG, periaqueductal gray; Pn, pontine nuclei; Pt, parietal cortex; Rn, raphe nuclei; RS, retrosplenial cortex; S1, primary somatosensory cortex; SN, substantia nigra; Sub, subiculum; Th, thalamus

To demonstrate the longitudinal changes in PAG FC pattern during the course of MIA‐induced OA progression, we investigated which brain regions had increased FC with PAG in the early phase compared to the late phase of OA in groups stratified by sex and age. YM had increased PAG FC with thalamus, caudate putamen, insula, hypothalamus, deep mesencephalic nucleus, anterior pretectal nucleus, entorhinal cortex, hippocampus, substantia nigra, retrosplenial cortex, inferior colliculus, and subiculum in the early phase relative to the late phase (Figure 4a). YF had increased PAG FC with caudate putamen, NAc, primary somatosensory cortex, thalamus, parietal cortex, deep mesencephalic nucleus, retrosplenial cortex, and hippocampus in the early phase relative to the late phase (Figure 4b). OM had increased PAG FC with hypothalamus, retrosplenial cortex, hippocampus, primary and secondary somatosensory cortices, thalamus, entorhinal cortex, pontine nuclei, and raphe nuclei in the early phase relative to the late phase (Figure 4c). OF had increased PAG FC with thalamus, caudate putamen, amygdala, insula, hippocampus, retrosplenial cortex, parietal cortex, entorhinal cortex, and deep mesencephalic nucleus in the early phase relative to the late phase (Figure 4d). Interestingly, all groups stratified by sex and age show greater PAG FC changes during the early phase compared to the late phase of OA. There are common brain regions that show increased FC with PAG during the early phase in all groups: thalamus, hippocampus, and retrosplenial cortex. Some particularities are also seen in PAG FC patterns of the different groups. The increased PAG FC with NAc was observed only in YF, while the increased PAG FC with amygdala was observed only in OF. Anterior pretectal nucleus and substantia nigra had increased FC with PAG only in YM, while the raphe nuclei had increased FC with PAG only in OM. These results support the importance of sex‐ and age‐matched groups to investigate brain mechanisms of OA pain.

### 2.5 Enhanced FC of PAG in the late phase of MIA‐induced OA in groups stratified by sex and age

**FIGURE 5:**
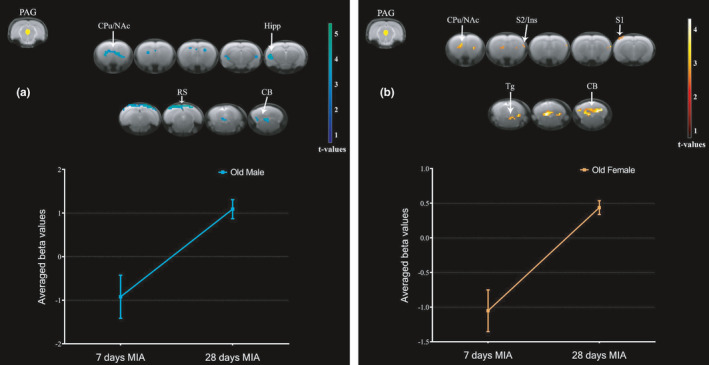
Increased PAG connectivity to the whole brain in the late phase of MIA‐induced OA pain in groups stratified by sex and age. Increased connectivity between PAG and brain regions at 28 days compared to 7 days after MIA injection in old males (a) and old females (b). We used a cluster‐forming threshold of *p* < 0.05, and t‐values are represented using the color‐coded scales. Plots show extracted beta values from all significant clusters for each group (average ±S.E.M.). CB, cerebellum; CPu, caudate putamen; Hipp, hippocampus; Ins, insula; NAc, nucleus accumbens; PAG, periaqueductal gray; RS, retrosplenial cortex; S1, primary somatosensory cortex; S2, secondary somatosensory cortex; Tg, tegmental nucleus

To further demonstrate the longitudinal changes in PAG FC pattern during the course of MIA‐induced OA progression, we investigated which brain regions had increased FC with PAG in the late phase compared to the early phase of OA in groups stratified by sex and age. As expected, young groups did not show increases in PAG FC in the late phase. This finding may be associated with the faster recovery of the pain behavior after MIA‐induced OA model in young groups compared to old groups (Ro et al., 2020). MIA induces more pronounced and longer‐lasting hyperalgesia in OF and OM than in sex‐matched young rats (Ro et al., 2020). We found that OM had increased PAG FC with caudate putamen, hippocampus, retrosplenial cortex, and cerebellum in the late phase relative to the early phase (Figure 5a). OF had increased PAG FC with caudate putamen, NAc, primary and secondary somatosensory cortices, insula, tegmental nucleus, and cerebellum in the late phase relative to the early phase (Figure 5b). OM continued to show increased PAG FC with hippocampus and retrosplenial in the late phase of OA. In contrast, OF had increased PAG FC with primary and secondary somatosensory cortices and tegmental nucleus in the late phase of OA, which was not seen in the early phase. The increased connectivity between PAG, caudate putamen, and NAc in both older groups shows that these regions may be relevant for brain processing of chronic OA pain.

## 3 DISCUSSION

We previously reported that intra‐articular injection of MIA induces more pronounced and longer‐lasting hyperalgesia in old rats than in young rats, with aged female rats exhibiting the most impaired responses (Ro et al., 2020). The present study defines a brain mechanism, particularly involving the PAG, for the roles of sex and age in acute and chronic phases of OA. Furthermore, the results of this study identify potential areas for circuit manipulations to experimentally demonstrate the causal relationships between OA pain and the PAG network, which may ultimately yield novel insights for personalized OA therapy.

**FIGURE 6:**
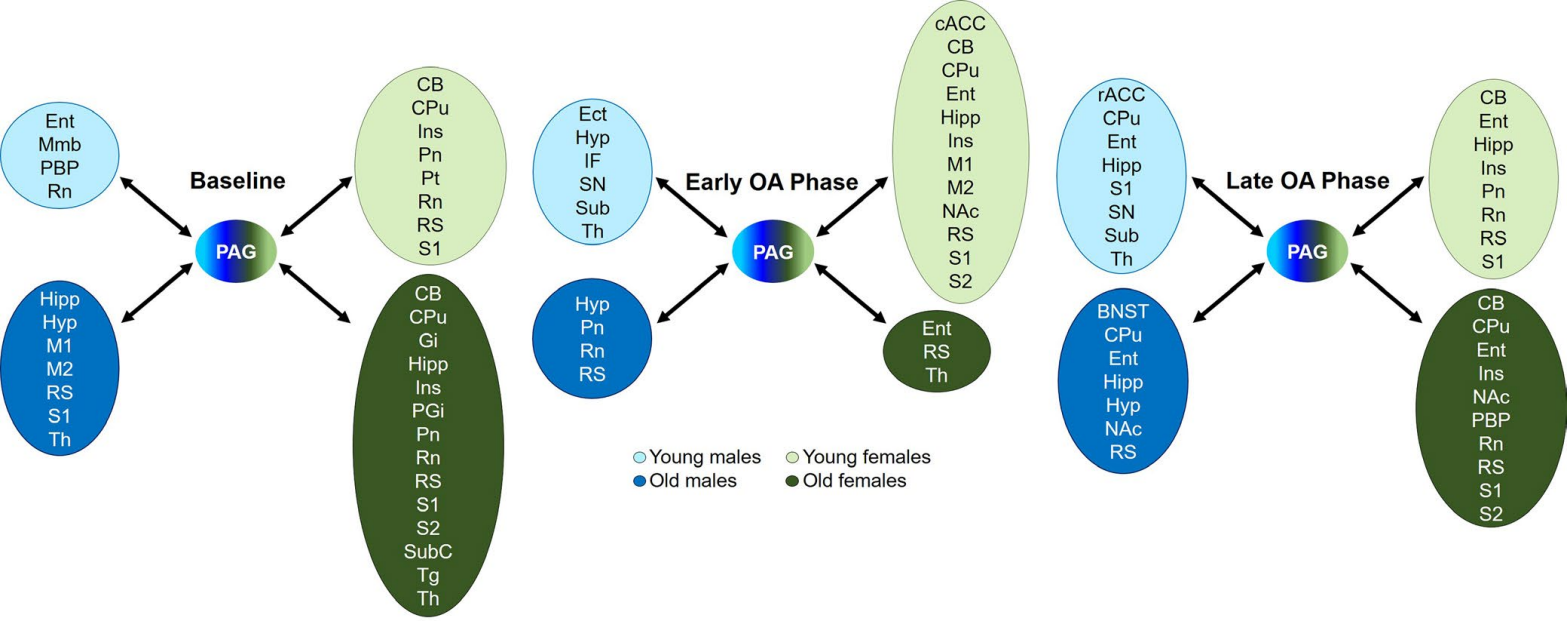
Model of potential pathways related to sex and age differences in PAG connectivity in a healthy state (baseline) and in the early and late phases of MIA‐induced OA. Group comparisons exhibited in Figures 1, 2, 3 were diagrammatically represented to show brain regions that had increased connectivity with PAG (ROI) at different time points. Young males, the least vulnerable OA group, exhibit moderate changes in PAG connectivity at all time points. Young females appear to recruit a more widespread PAG network in the early phase of OA. Older groups have stronger PAG connectivity with few brain regions in the early phase of OA. In contrast, the recruitment of additional brain regions in the late phase appears to contribute to the extended duration and high intensity of OA pain behavior in older groups. cACC, caudal anterior cingulate cortex; BNST, bed nucleus of the stria terminalis; CB, cerebellum; CPu, caudate putamen; Ect, ectorhinal cortex; Ent, entorhinal cortex; Gi, gigantocellular reticular nucleus; Hipp, hippocampus; Hyp, hypothalamus; IF, inferior colliculus; Ins, insula; M1, primary motor cortex; M2, secondary motor cortex; Mmb, mammillary body; NAc, nucleus accumbens; PAG, periaqueductal gray; PBP, parabrachial pigmented nucleus of the ventral tegmental area; PGi, paragigantocellular nucleus; Pn, pontine nuclei; Pt, parietal cortex; rACC, rostral anterior cingulate cortex; Rn, raphe nuclei; RS, retrosplenial cortex; S1, primary somatosensory cortex; S2, secondary somatosensory cortex; SN, substantia nigra; Sub, subiculum; SubC, subcoeruleus nucleus; Tg, tegmental nucleus; Th, thalamus

Based on our results, we constructed a model showing the sex and age differences in PAG FC in a healthy state and in the early and late phases of MIA‐induced OA (Figure 6). PAG connectivity pattern is influenced by sex and age in a healthy state and throughout the OA progression. Young males, the least vulnerable OA group, exhibit moderate changes in PAG FC compared to other groups. Young females appear to recruit a more widespread PAG network in the early phase of OA, including connections to NAc, which may represent a mechanism underlying the increased risk for females of developing chronic pain. Additionally, cACC and rACC regions may play distinct roles in the OA progression in YFs and males, delaying or accelerating the pain recovery respectively (Schweinhardt & Bushnell, 2010; Tan et al., 2017). Older groups have stronger PAG FC with fewer brain regions in the early phase of OA compared to the baseline (healthy state). In contrast, the recruitment of additional brain regions and the increased PAG FC with NAc in the late phase appear to contribute to the extended duration and high intensity of OA pain behavior in older groups.

Despite vast literature on the role of PAG in pain modulation, our knowledge of the influences of sex and age on PAG FC in healthy and chronic pain conditions is still limited (Coulombe et al., 2016; Da Silva et al., 2020; González‐Roldán et al., 2020). Our previous studies have shown that endogenous pain inhibition is impaired in healthy older rats of both sexes and is accompanied by changes in several brain circuitries, including PAG connections (Da Silva et al., 2018, 2020). In a healthy state and without any stimulus application, we found that the pattern of PAG FC with the whole brain is different between groups stratified by sex and age. Young females showed increased PAG FC with cerebellum and cortical regions including insula and primary somatosensory and parietal cortices, which was consistent with previous observations in healthy young women (Coulombe et al., 2016). Compared to men, women have greater PAG FC with cerebellum and cortical areas, such as insula, inferior frontal gyrus, orbitofrontal cortex, ACC, and premotor cortex during resting state (Coulombe et al., 2016). Sex differences are also observed in PAG FC modulated by exposure to low versus high experimental pain (Linnman et al., 2012). Healthy young men displayed a pain‐induced increase in PAG FC with the amygdala, caudate, and putamen that is not observed in women (Linnman et al., 2012). Our findings not only confirm that there are sex differences in PAG connectivity in a healthy state, but also show that age appears to exaggerate these sex differences. The female sex appears to contribute to the most widespread PAG connectivity pattern at baseline compared to age‐matched males. Even though both aged groups showed more widespread PAG FC with the whole brain at baseline, PAG FC pattern in OFs showed a greater number of engaged brain regions than OMs. Unfortunately, no studies have systemically assessed the effects of age on PAG FC in humans or animals when stratified by sex. However, healthy older individuals exhibit higher pain ratings to pressure stimulation and also exhibit altered connectivity between descending pain modulatory regions, including PAG, relative to younger adults (González‐Roldán et al., 2020). Thus, it is likely that distinct patterns of PAG networks in a healthy state can be associated with sex and age differences in pain responses and in brain dysregulation during pain conditions such as OA.

Interestingly, changes in PAG FC were greater in the early phase compared to the late phase of OA in all groups. An interpretation for these findings is that hyperalgesia, which is primarily driven by inflammation in the early phase of OA and is observed in all groups (Bove et al., 2003; Ro et al., 2020), may strongly alter how PAG connects to other brain regions in order to promote pain modulation. Furthermore, changes in PAG FC in the early phase also differed by sex and age. Although differences were modest between groups, YFs had increased PAG FC with basal ganglia, NAc, cACC, insula, and other cortical areas. Connections from cACC to insula can induce and maintain pain behavior by recruiting descending serotonergic facilitatory projections to the spinal cord (Tan et al., 2017). Thus, PAG connectivity changes in the early phase of OA may be associated with female's susceptibility to developing chronic pain (Fillingim, 2000; Larsson et al., 2017). Additionally, hyperalgesia in the early phase of MIA‐induced OA is greater in OFs than OM and young rats (Ro et al., 2020). OA pain is more prevalent and more widespread in older women than in older men (Leveille et al., 2005). Therefore, along with the evidence showing that young rats recover faster from MIA‐induced OA pain behavior than old rats (Ro et al., 2020), the PAG FC changes seen in the early phase may indicate a potential mechanism for the transition from acute to chronic OA pain and the increased risk for an aged population, especially women, of developing OA chronic pain (Fillingim, 2000).

Young Fisher rats exhibit primary mechanical hyperalgesia at the inflamed knee joint only during the early phase (7 days) of the MIA‐induced OA model, whereas old Fischer rats show profound primary mechanical hyperalgesia that persists during the late OA phase (Ro et al., 2020). Pain‐related responses in preclinical OA models may depend on rat strains, the dose of the chemicals used to induce OA, types of behavioral assays, and the site of stimulation (Burston et al., 2019; Fecho et al., 2005; Malfait et al., 2013; Yoon et al., 1999). For example, YM Sprague Dawley rats display secondary hyperalgesia at a cutaneous site remote from the inflamed knee joint that persists during the late phase (Burston et al., 2019), but it is not known how age or sex impacts primary hyperalgesia or any OA‐related pain responses in Sprague Dawley rats. Unfortunately, there is no study that directly compared MIA‐induced joint pathology or pain‐related responses between Fischer and Sprague Dawley rats. The use of Fisher rats in the present study allowed us to define clear sex and age differences in brain changes associated with OA progression, which was based on our previous study showing sex and age differences in MIA‐induced primary mechanical hyperalgesia (Ro et al., 2020).

Older males and females showed significantly increased connectivity between PAG, caudate putamen, and NAc in the late phase. The mesolimbic reward system, of which NAc is a major component, also plays a role in pain modulation (Gear et al., 1999; Tobaldini et al., 2019). Opioid and dopamine antagonists injected into NAc block endogenous pain inhibitory responses in rats (Gear et al., 1999; Tobaldini et al., 2019). Interestingly, exercise and mind‐body interventions reduce OA pain and modulate FC of the descending opioidergic pathway and reward system in patients with knee OA (Liu et al., 2019).Thus, the enhanced PAG FC with reward processing regions (Liu et al., 2019), as well as the predisposition to impaired descending pain inhibition (Da Silva et al., 2020) and the persistent hyperalgesia in the late phase (Ro et al., 2020), appears to contribute to the mechanisms underlying chronic OA pain in the elderly population and reveal potential strategies to treat OA pain. In contrast, findings from the late phase in young rats may potentially elucidate some aspects of the pain sensitivity recovery from the MIA model (Ro et al., 2020). Young males showed increased PAG FC with rACC in the late phase. A cortical top‐down modulatory pathway involves descending input from the rACC that can directly or indirectly arrive at PAG and mediate pain inhibition through opioidergic transmission (Schweinhardt & Bushnell, 2010). Young males exhibit stronger rACC‐PAG connectivity during DNIC relative to YFs and old rats (Da Silva et al., 2018, 2020). These observations and our data indicate that an increased rACC‐PAG connectivity could be responsible, at least in part, for the fast recovery from the OA pain observed in YMs (Ro et al., 2020). Even though YFs only exhibit OA pain‐related behavior for 14 days in the MIA model (Ro et al., 2020), their recovery is slower than that of YMs and may be associated with the widespread PAG network observed in the late phase.

This study had some limitations that reduce the weight of the inferences drawn from these findings. First, we used seed‐based analysis, which computes the cross‐correlation between the time series of the seed (PAG) and the rest of the brain. The coupling of activation between different brain areas indicates that they are involved in the same underlying functional process and are thus interpreted as functionally connected (Lv et al., 2018). The connectivity between the PAG and brain regions in this study can be the result of a direct anatomical connection or an indirect path via PAG, leading us to examine the overall organization of the PAG network and not the role of particular connections (Lv et al., 2018; van den Heuvel & Hulshoff Pol, 2010). Second, we did not perform functional MRI (fMRI) scans in control groups. Our previous behavioral study showed that the sex‐ and age‐matched control groups that received intra‐articular injection of saline did not develop impaired weight‐bearing responses or knee joint sensitivity (Ro et al., 2020). Third, anesthesia can modulate the FC of the brain. We used a low dose of isoflurane during the fMRI scans that is widely employed in in vivo electrophysiology studies from the pain field (Cummins et al., 2020; Dickenson et al., 2020). The exposure to isoflurane during the scan was also shorter than standard in vivo techniques and did not require tracheal intubation. Resting‐state networks are generally preserved and highly reproducible between animals under low dose of isoflurane compared to alpha‐chloralose and awake fMRI protocol, which can significantly induce stress (Da Silva & Seminowicz, 2019; Low et al., 2016; Williams et al., 2010).

In summary, our study provides insight into the impact of sex and age on pain modulatory networks in a healthy state and throughout OA progression. Our findings suggest that distinct patterns of PAG FC seen in groups stratified by sex and age may contribute to ultimately shaping the pain responses and recovery during OA progression in a sex‐ and age‐dependent manner. Stronger rACC‐PAG connectivity in YMs during the late phase may facilitate the descending pain inhibitory network and thus render YMs the least vulnerable group for developing chronic pain conditions. Our findings also suggest that a widespread PAG network and the participation of regions related to reward processing seem to enhance the elderly's susceptibility to chronic OA pain, particularly for the female sex. Therefore, therapeutic strategies that strengthen the pain inhibitory system may represent a potential avenue of early pain management to help prevent chronic OA pain.

## 4 EXPERIMENTAL PROCEDURES

### 4.1 Animals and ethical approval

Male and female Fischer 344 rats of young (3–6 months old) and old (20–24 months old) age were obtained from the National Institute on Aging. All animals were housed in a temperature‐controlled room under a 12:12‐h light–dark cycle with access to food and water ad libitum. All imaging scans were conducted in the morning (between 8:00 and 11:30 AM). The animals were monitored throughout the duration of the study to reduce unnecessary stress and/or pain. All procedures were conducted in accordance with the National Institutes of Health Guide for the Care and Use of Laboratory Animals and under a University of Maryland‐approved Institutional Animal Care and Use Committee protocol. There were no animals missing from analysis; all rats survived the intra‐articular injections and experimental protocol. We did not exclude any outliers from the analysis. Sample size (*n* = 8) was determined based on previous publications and power analyses (expected mean: 50% of population mean, power: 0.8, α level: 0.05) (Da Silva et al., 2020; Ro et al., 2020).

### 4.2 Drug preparation and administration

Monoiodoacetate (MIA—Sigma‐Aldrich) was dissolved in sterile saline. A single intra‐articular injection of MIA (3 mg/50 μl saline) was made through the infra‐patellar ligament of the left knee using a 30‐gauge needle (Ro et al., 2020). Rats were anesthetized with isoflurane (1.5%–2%) for all injection procedures.

### 4.3 fMRI data acquisition

Data were acquired using a Bruker BioSpec 70/30USR Avance III 7‐Tesla scanner (Bruker Biospin MRI GmbH) and a 40‐mm circular polarized volume coil. During scanning, rats were anesthetized with a constant mixture of 1.5% isoflurane in oxygen‐enriched air, and respiration and heart rate were monitored with a small animal monitoring and gating system and software (SA Instruments, Inc.). T2‐weighted anatomical images were obtained using a 2D RARE (400 × 400 matrix, 22 coronal 1 mm slice thickness, in plane resolution 100 µm, TR 2000 ms, TE 28 ms). An rsfMRI scan was acquired using an echo planar imaging sequence (TR 1500 ms, TE 24 ms, 128 × 128 matrix, in plane resolution 0.40 × 0.40 × 1 mm, 22 coronal slices, 620 volumes per scan). The anatomical and rsfMRI scans were performed for each rat at baseline (healthy state) and in the early (7 days) and late (28 days) phases of MIA‐induced OA model. Thus, we performed 96 MRI sessions in total, since we used 32 rats (*n* = 8) and scanned each 3 times. We chose the time points after the MIA injection based upon prior literature showing sex and age differences in pain behavior and based upon distinct peripheral mechanisms between the early and late phases of the MIA‐induced OA model (Bove et al., 2003; Mapp et al., 2013; Ro et al., 2020). The investigators analyzing the MRI data were blinded to the experimental groups. Investigators running the MRI sessions were not blinded to the experimental groups due to visible sex and weight differences between animals.

### 4.4 fMRI preprocessing, statistical analysis, and data availability

All preprocessing and analyses were performed in SPM12 (http://www.fil.ion.ucl.ac.uk/spm/). We used seed‐based analysis to assess how FC between the PAG and the whole brain varies between groups and time points. We selected PAG as the region of interest (ROI) based upon prior literature showing its involvement in descending pain inhibition (Fields, 2004), which is also modulated by sex and age (Da Silva et al., 2018, 2020). Anatomical location was chosen according to Paxinos and Watson atlas (5th edition, 2004). We first created a study‐specific template by coregistering and averaging the T2‐weighted images across animals and interpolating to voxel size of isotropic 0.5 mm. Preprocessing steps included slice timing correction (number of slices: 22, reference slice: 11), realignment, and motion correction (separation: 0.57, smoothing—FWHM: 0.76, interpolation for reslicing: 4th Degree B‐Spline), within‐subject registration (separation: 1 0.5, histogram smoothing: 1.7 1.7), normalization to the study‐specific template (source imaging smoothing: 1.52), normalization of functional images (0.5 mm isotropic voxels, interpolation: trilinear), bandpass filtering (0.009–0.2 Hz), and smoothing at 1 mm FWHM. We then extracted time series data from the ROI and regressed this time course with the signal at each voxel across the whole brain to reveal FC patterns for each animal. Six motion parameters were included as regressors of no interest.

The second level (group) analyses used to address our hypothesis were the following: (1) Two‐Way ANOVA with sex and age as main factors and comparisons between the four groups to show sex and age differences in PAG FC with the whole brain separately at baseline, early OA phase, and late OA phase. (2) One‐Way ANOVA within‐subjects to show brain regions with increased and decreased FC with PAG in the early OA phase compared to the late OA phase in each group. In addition, we used two‐Way ANOVA for baseline, early OA phase, and late OA phase with sex and age as main factors (reported in supplementary material), to examine the main effects of sex (combined young and old groups) and age (combined males and females), as well as the sex effects in age‐matched groups (young rats and old rats separately) and age effects in sex‐matched groups (males and females separately).

Second level maps used cluster‐forming thresholds at *p* < 0.05, 0.01, and 0.005 for group comparisons, and all significant clusters were illustrated on the brain template. We used a cluster‐extent based thresholding approach due to its relatively high sensitivity to account for the fact that individual voxel activations are not independent of their neighboring voxels’ activations and as a correction method for multiple comparisons between all voxels in the brain (Smith & Nichols, 2009; Woo et al., 2014). Different cluster‐forming thresholds were reported to show the results from liberal thresholds commonly used in rodent fMRI studies (Zhao et al., 2012, 2014) as well as more stringent thresholds (Mathur et al., 2016; Woo et al., 2014). Second level maps used cluster‐forming thresholds at *p* < 0.05 for within‐subject comparisons since more stringent thresholds did not show time points’ differences, and all significant clusters were illustrated with their respective t‐values on the brain template. For visualization of the BOLD signal, we extracted and plotted the average beta values ±S.E.M. from all significant clusters at *p* < 0.05 for each animal and time point using MarsBaR (SPM toolbox) and GraphPad Prism 8.12. All data including code, ROI, and the template brain are available upon request and in NeuroVault (Da Silva, 2019).

## CONFLICTS OF INTEREST

All authors declare that they have no conflicts of interest.

## AUTHOR CONTRIBUTIONS

All authors were involved with drafting the article or revising it critically for important intellectual content and all authors approved the final version to be published. Dr. Da Silva had full access to all of the data and takes responsibility for the integrity of the data and the accuracy of the data analysis.

Da Silva, Ro, and Seminowicz **involved in study conception and design**. Da Silva, Tricou, Zhang, and Tofighbakhsh **involved in acquisition of data**. Da Silva, Ro, and Seminowicz **involved in analysis and interpretation of data**.

## Supporting information

Figures S1–S2

## Data Availability

All data including code, ROI, and the template brain are available in NeuroVault (Da Silva, 2019) and upon request.
