# Supplementary material for: Pain modulatory network is influenced by sex and age in a healthy state and during osteoarthritis progression in rats
Source: Aging Cell. 2021 Jan 5;20(2):e13292. doi: 10.1111/acel.13292 (PMC7884031; doi:10.1111/acel.13292)

**SUPPLEMENTARY METHODS AND RESULTS**

**Secondary analysis.**

We used Two-Way ANOVA for baseline, early OA phase, and late OA phase with sex and age as main factors to examine the main effects of sex (combined young and old groups, fig. S1A) and age (combined males and females, fig. S2A), as well as the sex effects in age-matched groups (young rats and old rats separately, fig. S1B and C), and age effects in sex-matched groups (males and females separately, fig. S2B and C).

**Supplementary figure 1.** Sex effects regardless of age (A) and sex effects in age-matched groups (B and C). Brain templates show cluster-forming threshold at p < 0.01.


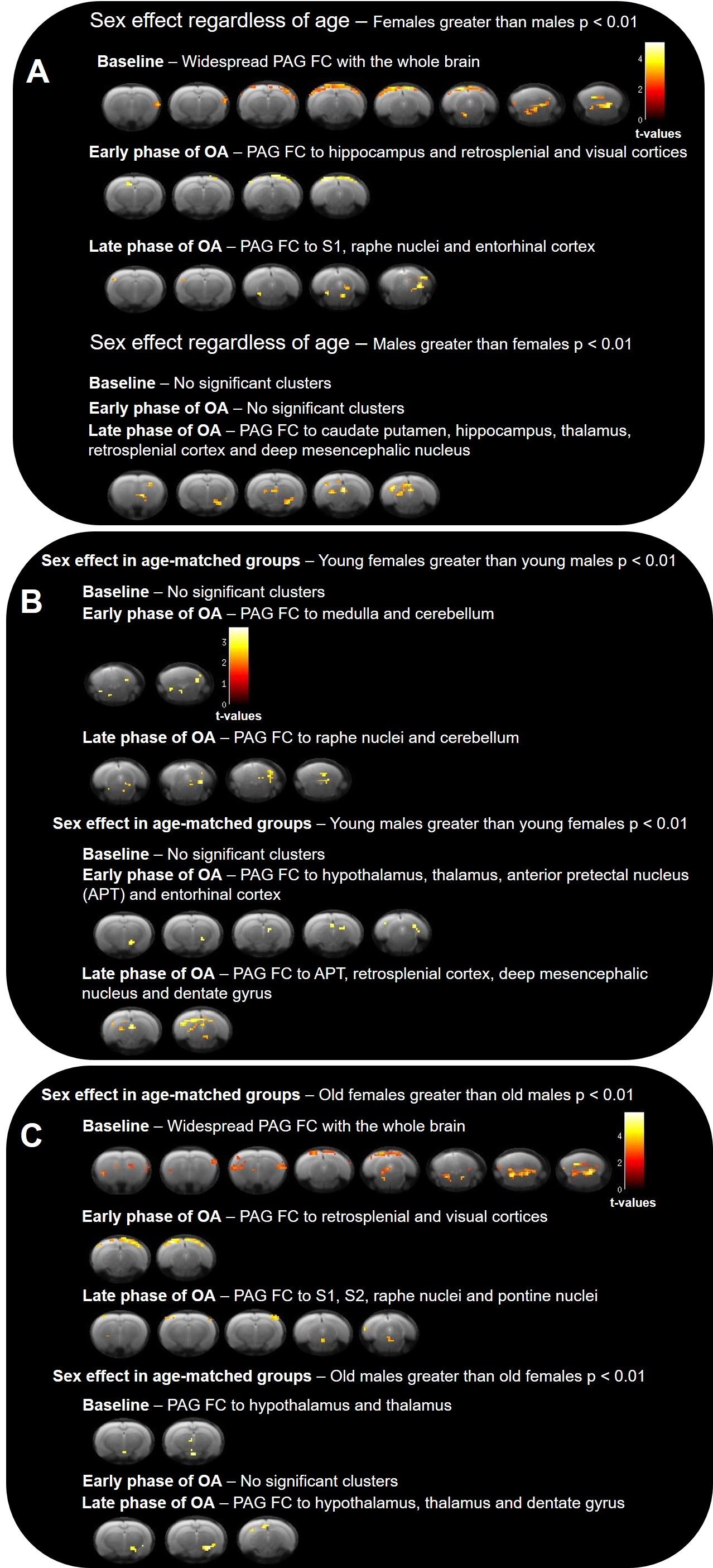


**Supplementary figure 2.** Age effects regardless of sex (A) and age effects in sex-matched groups (B and C). Brain templates show cluster-forming threshold at p < 0.01.


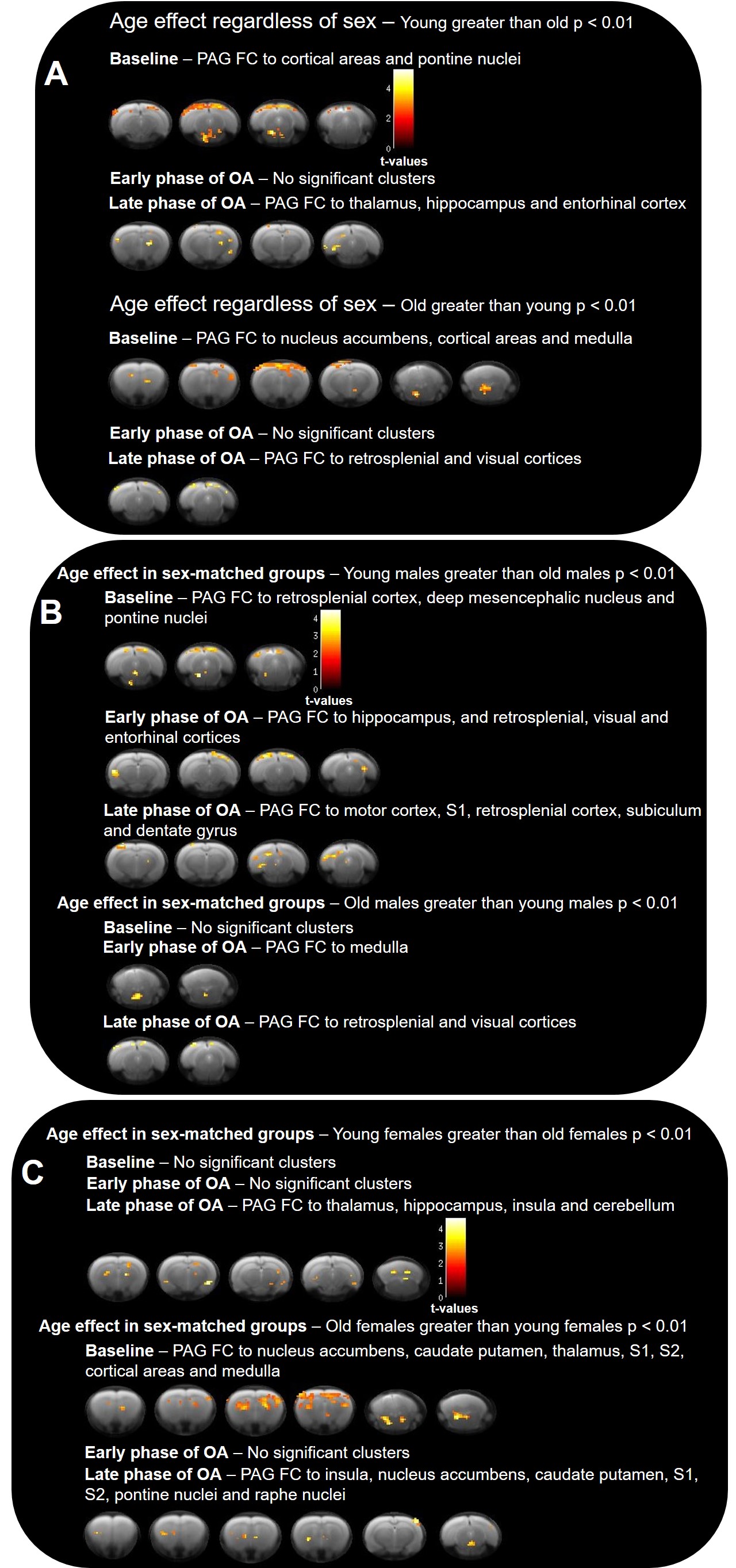

Supplement: Supplementary file 1 — Figures S1–S2 [file ACEL-20-e13292-s001.docx]
